# Supplementary material for: Comparison of Pretraining Models and Strategies for Health-Related Social Media Text Classification
Source: Healthcare (Basel). 2022 Aug 5;10(8):1478. doi: 10.3390/healthcare10081478 (PMC9408372; doi:10.3390/healthcare10081478)
Supplement: Supplementary file 1 [file healthcare-10-01478-s001.zip › Supplementary material.pdf]

SUPPLEMENTARY MATERIAL

Each of RoBERTa, BERTweet, and BioBERT has multiple implementations which differ in the model size, the pre-training data size, and the training time. The specific models for RoBERTa, BERTweet and BioBERT we used in our experiments are RoBERTa_Base, BERTweet_Base and BioBERT v1.1.

Table S1. The full hyper-parameter configurations of the classification model. The maximum sequence size of each model is set as the same as the off-the-shelf model, which is 128 for BERTweet and 512 for the rest models.

**Hyper-parameter**	
Number of Layers	12
Hidden size	768
FFN inner hidden size	3072
Attention heads	12
Attention head size	64
Dropout	0.1
Attention Dropout	0.1
Weight Decay	0.01
Learning Rate Decay	Linear
Adam β_1	0.9
Adam β_2	0.98
Gradient Clipping	0
Batch Size	32
Warmup Ratio	0
Adam Epsilon	1×10^{-8}

Table S2. The links to access the datasets. For Task 2-3, 7-12 and 15-16, the datasets are not publicly available but can be accessed by sending requests to the dataset creators.

ID	Task	Link
1	ADR Detection	http://diego.asu.edu/Publications/ADRClassify.html
2	Breast Cancer	https://healthlanguageprocessing.org/smm4h-2021/task-8/
3	NPMU characterization	-
4	WNUT-20-task2 (informative COVID-19 tweet detection)	https://github.com/VinAIResearch/COVID19Tweet
5	SMM4H-17-task1 (ADR detection)	https://healthlanguageprocessing.org/sharedtask2/
6	SMM4H-17-task2 (medication consumption)	

7	SMM4H-21-task1 (ADR detection)	https://healthlanguageprocessing.org/smm4h-2021/task-1/
8	SMM4H-21-task3a (regimen change on Twitter)	https://healthlanguageprocessing.org/smm4h-2021/task-3/
9	SMM4H-21-task3b (regimen change on WebMD)	
10	SMM4H-21-task4 (adverse pregnancy outcomes)	https://healthlanguageprocessing.org/smm4h-2021/task-4/
11	SMM4H-21-task5 (COVID-19 potential case)	https://healthlanguageprocessing.org/smm4h-2021/task-5/
12	SMM4H-21-task6 (COVID-19 symptom)	https://healthlanguageprocessing.org/smm4h-2021/task-6/
13	Suicidal Ideation Detection	https://zenodo.org/record/4278895#.YT_KL1P0kWo
14	Drug Addiction and Recovery Intervention	https://zenodo.org/record/4543776#.YUJW-FP0kWo
15	eRisk-21-task1 (Signs of Pathological Gambling)	https://early.irlab.org/
16	eRisk-21-task2 (Signs of Self-Harm)	
17	Sentiment Analysis in e-Health Forums (Food Allergy Related)	https://zenodo.org/record/1479354#.YVHTeFP0kWo
18	Sentiment Analysis in e-Health Forums (Crohn'S Disease Related)	
19	Sentiment Analysis in e-Health Forums (Breast Cancer Related)	
20	Factuality Classification in e-Health Forums (Food Allergy Related)	
21	Factuality Classification in e-Health Forums (Crohn'S Disease Related)	
22	Factuality Classification in e-Health Forums (Breast Cancer Related)	
